# Supplementary material for: Effect of Al Layer Thickness on the Bonding and Mechanical Behavior of a Mg-(Al-)Ti Laminated Sheet Prepared by Hot-Rolling after Differential Preheating Treatment
Source: Materials (Basel). 2022 Apr 11;15(8):2805. doi: 10.3390/ma15082805 (PMC9024959; doi:10.3390/ma15082805)
Supplement: Supplementary file 1 [file materials-15-02805-s001.zip › materials-1630793-supplementary.pdf]

Table S1 The original nanoindentation data for matrix scanning.

| Point | hc(nm) | Pmax(μN) | S(μN/nm) | A(nm^2) | hmax(nm) | heff(nm) | Er(GPa) | H(GPa) | A | hf(nm) |
|---|---|---|---|---|---|---|---|---|---|---|
| 1 | 338.3409 | 1998.366 | 168.2056 | 4.85E+06 | 347.3316 | 347.2513 | 67.67995 | 0.41214 | 0.80524 | 319.3518 |
| 2 | 271.9669 | 1998.279 | 121.5578 | 3.36E+06 | 284.2048 | 284.2961 | 58.79017 | 0.59543 | 0.12657 | 241.8884 |
| 3 | 319.1257 | 1998.264 | 88.15518 | 4.39E+06 | 333.9969 | 336.1264 | 37.27377 | 0.45509 | 0 | 222.7885 |
| 4 | 265.0684 | 1998.403 | 143.639 | 3.22E+06 | 275.5318 | 275.5029 | 70.97331 | 0.62153 | 0.00199 | 226.1849 |
| 5 | 196.9628 | 1998.165 | 123.8944 | 1.98E+06 | 208.8671 | 209.0588 | 78.09383 | 1.01133 | 3.74217 | 179.2287 |
| 6 | 153.4022 | 1998.306 | 127.131 | 1.33E+06 | 164.9382 | 165.1911 | 97.68564 | 1.50297 | 4.96891 | 136.9698 |
| 7 | 163.8505 | 1998.299 | 139.687 | 1.47E+06 | 174.3439 | 174.5796 | 101.9449 | 1.35584 | 13.26022 | 151.6691 |
| 8 | 291.0545 | 1998.203 | 138.663 | 3.76E+06 | 301.1019 | 301.8624 | 63.36104 | 0.53149 | 2.00E-06 | 231.1789 |
| 9 | 328.8848 | 1998.363 | 119.4993 | 4.62E+06 | 340.2877 | 341.4269 | 49.25361 | 0.43246 | 0 | 257.8129 |
| 10 | 295.3212 | 1998.065 | 133.0096 | 3.85E+06 | 306.5237 | 306.5877 | 60.03919 | 0.51861 | 0.11765 | 266.8527 |
| 11 | 222.578 | 1998.214 | 140.1798 | 2.41E+06 | 233.3652 | 233.269 | 80.00793 | 0.82922 | 0.0077 | 186.9418 |
| 12 | 267.5752 | 1998.218 | 128.812 | 3.27E+06 | 279.1204 | 279.2097 | 63.15014 | 0.6118 | 0.60554 | 243.9375 |
| 13 | 277.584 | 1998.407 | 134.7634 | 3.47E+06 | 288.7037 | 288.7057 | 64.07337 | 0.57547 | 1.15959 | 256.7947 |
| 14 | 321.1229 | 1998.38 | 123.2795 | 4.44E+06 | 333.2719 | 333.2806 | 51.85052 | 0.45033 | 10.04909 | 307.0264 |
| 15 | 337.5994 | 1998.348 | 144.4223 | 4.83E+06 | 348.0423 | 347.9771 | 58.21886 | 0.41368 | 0.03832 | 307.3938 |
| 16 | 294.0651 | 1998.311 | 137.1204 | 3.83E+06 | 304.9811 | 304.9951 | 62.11692 | 0.52241 | 1.61302 | 274.5969 |
| 17 | 244.4948 | 1998.27 | 126.437 | 2.81E+06 | 256.5521 | 256.3482 | 66.80306 | 0.71061 | 5.07695 | 228.0864 |
| 18 | 326.0436 | 1998.344 | 130.8915 | 4.55E+06 | 336.9368 | 337.4939 | 54.34748 | 0.43887 | 1.00E-06 | 261.1581 |
| 19 | 287.3718 | 1998.267 | 146.1301 | 3.68E+06 | 297.5103 | 297.6277 | 67.49046 | 0.54299 | 2.90E-05 | 237.4378 |
| 20 | 218.1081 | 1998.347 | 120.6305 | 2.33E+06 | 230.52 | 230.5324 | 70.00059 | 0.85722 | 14.23493 | 205.1926 |
| 21 | 252.0275 | 1998.425 | 137.2734 | 2.96E+06 | 263.0083 | 262.946 | 70.73047 | 0.67586 | 3.53822 | 235.1898 |
| 22 | 257.7365 | 1998.357 | 108.9748 | 3.07E+06 | 270.5016 | 271.4898 | 55.11597 | 0.65119 | 0.01366 | 216.9514 |

| | | | | | | | | | | |
|---|---|---|---|---|---|---|---|---|---|---|
| 23 | 200.6765 | 1998.331 | 140.1503 | 2.04E+06 | 211.3198 | 211.3703 | 87.01864 | 0.98137 | 3.59465 | 184.1065 |
| 24 | 321.5001 | 1998.254 | 167.4757 | 4.45E+06 | 330.495 | 330.4488 | 70.36918 | 0.44941 | 0.54097 | 301.3665 |
| 25 | 309.5141 | 1998.243 | 89.0718 | 4.17E+06 | 322.8701 | 326.3397 | 38.64734 | 0.47922 | 0 | 214.1693 |
| 26 | 225.6399 | 1998.39 | 127.7199 | 2.46E+06 | 237.3579 | 237.3749 | 72.08585 | 0.81095 | 13.95022 | 213.0389 |
| 27 | 232.5612 | 1998.239 | 81.86563 | 2.59E+06 | 247.3997 | 250.8678 | 45.07424 | 0.77167 | 4.20E-04 | 166.2939 |
| 28 | 259.8301 | 1998.325 | 82.79823 | 3.11E+06 | 274.7394 | 277.9312 | 41.59624 | 0.64248 | 0.00289 | 202.7905 |
| 29 | 267.9139 | 1998.32 | 79.85332 | 3.27E+06 | 282.25 | 286.6826 | 39.10687 | 0.61054 | 7.36E-04 | 202.9493 |
| 30 | 240.6118 | 1998.377 | 153.6989 | 2.74E+06 | 250.6199 | 250.3632 | 82.28697 | 0.72967 | 0.2092 | 216.5267 |
| 31 | 206.1946 | 1998.406 | 122.9663 | 2.13E+06 | 218.3263 | 218.3833 | 74.69141 | 0.93925 | 11.43641 | 192.571 |
| 32 | 334.1103 | 1998.369 | 133.1499 | 4.75E+06 | 345.31 | 345.3666 | 54.15062 | 0.42105 | 5.09659 | 318.2188 |
| 33 | 173.091 | 1998.253 | 128.4705 | 1.61E+06 | 184.6321 | 184.7566 | 89.78712 | 1.24337 | 25.38181 | 162.7794 |
| 34 | 264.826 | 1998.437 | 135.5778 | 3.21E+06 | 276.071 | 275.8811 | 67.04126 | 0.62248 | 2.30401 | 246.4075 |
| 35 | 266.955 | 1998.397 | 81.23314 | 3.25E+06 | 280.7606 | 285.4056 | 39.90165 | 0.61423 | 9.04E-04 | 203.7687 |
| 36 | 237.6115 | 1998.391 | 174.7931 | 2.68E+06 | 246.3292 | 246.1862 | 94.5531 | 0.74493 | 5.46723 | 224.3167 |
| 37 | 366.0271 | 1998.363 | 111.2338 | 5.54E+06 | 377.7697 | 379.5012 | 41.85358 | 0.36041 | 0 | 289.674 |
| 38 | 283.0447 | 1998.343 | 126.0166 | 3.59E+06 | 295.1304 | 294.938 | 58.94584 | 0.55699 | 15.19785 | 270.6761 |
| 39 | 266.1057 | 1998.082 | 164.7456 | 3.24E+06 | 275.3343 | 275.2019 | 81.13801 | 0.6174 | 3.00E-05 | 220.5976 |
| 40 | 291.6482 | 1998.402 | 131.0615 | 3.77E+06 | 303.2629 | 303.0841 | 59.78524 | 0.52973 | 1.27412 | 270.7961 |
| 41 | 204.982 | 1998.293 | 129.1874 | 2.11E+06 | 216.7224 | 216.5831 | 78.84613 | 0.94822 | 9.40386 | 191.0108 |
| 42 | 298.6273 | 1998.16 | 128.4467 | 3.93E+06 | 310.3928 | 310.2946 | 57.43914 | 0.50902 | 16.42856 | 286.6754 |
| 43 | 239.2478 | 1998.116 | 83.85992 | 2.71E+06 | 252.884 | 257.118 | 45.10765 | 0.73645 | 6.63E-04 | 176.2081 |
| 44 | 228.7573 | 1998.314 | 116.9028 | 2.52E+06 | 240.4503 | 241.5777 | 65.24266 | 0.79288 | 0.03599 | 193.3875 |
| 45 | 238.8904 | 1998.448 | 112.7762 | 2.71E+06 | 250.9704 | 252.1808 | 60.7363 | 0.73839 | 0.02247 | 200.8935 |
| 46 | 198.958 | 1998.399 | 101.6008 | 2.01E+06 | 211.6362 | 213.7099 | 63.52311 | 0.99513 | 0 | 115.3642 |

| | | | | | | | | | | |
|---|---|---|---|---|---|---|---|---|---|---|
| 47 | 217.5297 | 1998.067 | 127.0582 | 2.32E+06 | 229.3852 | 229.3239 | 73.89044 | 0.86082 | 7.57497 | 202.6306 |
| 48 | 197.9419 | 1998.371 | 132.2626 | 1.99E+06 | 209.492 | 209.2738 | 83.03583 | 1.00337 | 12.8782 | 185.2868 |
| 49 | 297.6609 | 1998.172 | 158.4603 | 3.90E+06 | 307.0829 | 307.1183 | 71.05425 | 0.5118 | 0.74409 | 277.6815 |
| 50 | 231.2048 | 1998.265 | 133.7743 | 2.56E+06 | 242.4154 | 242.408 | 74.00929 | 0.77913 | 1.01612 | 209.8752 |
| 51 | 184.5439 | 1998.179 | 136.4556 | 1.78E+06 | 195.5761 | 195.5265 | 90.6275 | 1.1228 | 8.27927 | 170.5584 |
| 52 | 289.3097 | 1998.198 | 136.7725 | 3.72E+06 | 300.4451 | 300.2669 | 62.81346 | 0.53688 | 0.16474 | 262.4499 |
| 53 | 269.0912 | 1998.25 | 126.3162 | 3.30E+06 | 281.1592 | 280.9558 | 61.6356 | 0.60607 | 4.41403 | 252.1648 |
| 54 | 175.0224 | 1998.346 | 120.9324 | 1.64E+06 | 187.1425 | 187.4157 | 83.7785 | 1.22175 | 1.00E-05 | 113.89 |
| 55 | 203.2896 | 1998.117 | 141.6816 | 2.08E+06 | 213.5783 | 213.8668 | 87.05395 | 0.96095 | 2.00E-06 | 144.7579 |
| 56 | 214.1712 | 1998.341 | 120.9255 | 2.26E+06 | 226.4157 | 226.5652 | 71.22137 | 0.88305 | 2.19E-04 | 162.8203 |
| 57 | 126.8638 | 1998.314 | 104.7626 | 994296.8 | 141.1855 | 141.1698 | 93.08575 | 2.00978 | 7.42005 | 110.1053 |
| 58 | 234.9144 | 1998.296 | 124.4771 | 2.63E+06 | 246.9648 | 246.9546 | 67.97066 | 0.75902 | 13.83588 | 222.1066 |
| 59 | 186.046 | 1998.292 | 72.87086 | 1.80E+06 | 201.0625 | 206.6128 | 48.08472 | 1.1084 | 5.88E-04 | 115.2665 |
| 60 | 278.6836 | 1998.309 | 81.27504 | 3.50E+06 | 293.5468 | 297.1238 | 38.51473 | 0.57165 | 0.00352 | 221.7527 |
| 61 | 281.5817 | 1998.345 | 132.4946 | 3.56E+06 | 293.2042 | 292.8936 | 62.2455 | 0.56185 | 0.04457 | 249.9326 |
| 62 | 173.601 | 1998.283 | 124.2851 | 1.61E+06 | 186.0226 | 185.6597 | 86.65971 | 1.23761 | 0.04086 | 140.1801 |
| 63 | 113.4397 | 1998.231 | 98.68237 | 842084.7 | 128.4653 | 128.6266 | 95.27885 | 2.37296 | 8.42301 | 96.66243 |
| 64 | 119.6426 | 1998.295 | 105.678 | 910946 | 133.7636 | 133.8246 | 98.10089 | 2.19365 | 3.13367 | 99.33552 |
| 65 | 105.6158 | 1998.349 | 97.64211 | 758864.5 | 120.9315 | 120.9653 | 99.30928 | 2.63334 | 8.45061 | 88.75039 |
| 66 | 118.6362 | 1998.157 | 107.1996 | 899600.9 | 132.4927 | 132.6159 | 100.139 | 2.22116 | 9.33245 | 103.0726 |
| 67 | 115.4545 | 1998.327 | 106.8474 | 864173 | 129.4654 | 129.4815 | 101.8353 | 2.31242 | 13.95299 | 101.5865 |
| 68 | 165.502 | 1998.187 | 127.4849 | 1.50E+06 | 177.4044 | 177.2575 | 92.3088 | 1.33455 | 1.92561 | 145.7177 |
| 69 | 200.2649 | 1998.357 | 79.18381 | 2.03E+06 | 215.4581 | 219.1926 | 49.24647 | 0.98465 | 0.00144 | 138.0017 |
| 70 | 146.4523 | 1998.364 | 120.2211 | 1.24E+06 | 158.8682 | 158.9191 | 95.75425 | 1.61495 | 4.22239 | 128.839 |

| | | | | | | | | | | |
|---|---|---|---|---|---|---|---|---|---|---|
| 71 | 100.5582 | 1998.415 | 110.1857 | 707246.4 | 114.1857 | 114.1608 | 116.0846 | 2.82563 | 5.30987 | 82.91129 |
| 72 | 128.0326 | 1998.172 | 119.78 | 1.01E+06 | 140.6042 | 140.5441 | 105.6977 | 1.9821 | 3.16888 | 109.2988 |
| 73 | 132.2487 | 1998.119 | 111.3645 | 1.06E+06 | 145.7565 | 145.7053 | 95.89644 | 1.8874 | 4.42111 | 113.9772 |
| 74 | 116.1125 | 1998.213 | 101.0639 | 871445.6 | 130.8311 | 130.9414 | 95.92038 | 2.29299 | 10.89078 | 100.7106 |
| 75 | 97.94447 | 1998.305 | 102.8655 | 681244.9 | 112.5371 | 112.5143 | 110.4213 | 2.93331 | 7.25759 | 80.91025 |
| 76 | 104.2301 | 1998.349 | 100.6007 | 744551.6 | 119.102 | 119.1283 | 103.2972 | 2.68396 | 9.77365 | 88.3017 |
| 77 | 110.4134 | 1998.286 | 103.0168 | 809411.6 | 124.7745 | 124.9617 | 101.4514 | 2.46881 | 11.20691 | 95.30014 |
| 78 | 111.4022 | 1998.409 | 103.8723 | 820019.5 | 125.8765 | 125.8315 | 101.6302 | 2.43703 | 10.33357 | 96.00147 |
| 79 | 95.23695 | 1998.358 | 100.223 | 654797.4 | 110.2124 | 110.1913 | 109.7359 | 3.05187 | 9.72472 | 79.25211 |
| 80 | 172.03 | 1998.341 | 119.536 | 1.59E+06 | 184.6859 | 184.5681 | 83.95053 | 1.25559 | 0.54249 | 146.7731 |
| 81 | 145.5885 | 1998.207 | 111.317 | 1.23E+06 | 158.9611 | 159.0515 | 89.06764 | 1.62963 | 7.63276 | 129.5283 |
| 82 | 114.9486 | 1998.172 | 100.0852 | 858601.7 | 129.7453 | 129.9222 | 95.69927 | 2.32724 | 16.89098 | 101.4627 |
| 83 | 115.1952 | 1998.046 | 103.5318 | 861315.5 | 129.7474 | 129.6694 | 98.83884 | 2.31976 | 10.69575 | 99.91983 |
| 84 | 136.3729 | 1998.412 | 105.2954 | 1.11E+06 | 150.5968 | 150.6072 | 88.57943 | 1.80162 | 10.02245 | 120.9567 |
| 85 | 118.9213 | 1998.325 | 104.8863 | 902807.9 | 133.2137 | 133.2105 | 97.80386 | 2.21346 | 10.23687 | 103.5638 |
| 86 | 117.5615 | 1998.274 | 99.28129 | 887559.3 | 132.5941 | 132.657 | 93.36918 | 2.25143 | 12.75465 | 102.7273 |
| 87 | 108.0169 | 1998.151 | 101.9148 | 783970.7 | 122.7265 | 122.7215 | 101.9817 | 2.54876 | 7.30762 | 90.92151 |
| 88 | 108.3303 | 1998.237 | 99.44839 | 787275.9 | 123.2516 | 123.4002 | 99.30454 | 2.53817 | 15.29884 | 94.34359 |
| 89 | 140.9783 | 1998.071 | 109.0436 | 1.17E+06 | 154.7038 | 154.721 | 89.43361 | 1.71215 | 5.67804 | 123.4993 |
| 90 | 153.3266 | 1998.156 | 120.0009 | 1.33E+06 | 165.9823 | 165.815 | 92.24236 | 1.50401 | 0.9845 | 130.2952 |
| 91 | 110.2318 | 1998.169 | 109.8139 | 807470.2 | 124.1755 | 123.8788 | 108.2751 | 2.4746 | 0.94214 | 85.63197 |
| 92 | 121.8723 | 1998.364 | 101.7041 | 936318.7 | 136.6374 | 136.6089 | 93.12396 | 2.13428 | 12.07989 | 106.9865 |
| 93 | 110.6557 | 1998.182 | 109.3783 | 812005.2 | 124.4425 | 124.3571 | 107.5441 | 2.4608 | 7.97197 | 94.61169 |
| 94 | 105.2257 | 1998.142 | 103.371 | 754821.9 | 119.6622 | 119.723 | 105.4172 | 2.64717 | 11.61131 | 90.29828 |

| | | | | | | | | | | |
|---|---|---|---|---|---|---|---|---|---|---|
| 95 | 89.29164 | 1998.43 | 105.5156 | 598468.9 | 103.5617 | 103.4964 | 120.8455 | 3.33924 | 9.90225 | 73.84138 |
| 96 | 112.6347 | 1998.328 | 102.9371 | 833333.5 | 127.1591 | 127.1945 | 99.90734 | 2.39799 | 10.73325 | 97.32385 |
| 97 | 109.6898 | 1998.254 | 108.1294 | 801689.1 | 123.5608 | 123.5499 | 106.998 | 2.49255 | 8.79859 | 93.95332 |
| 98 | 140.321 | 1998.396 | 126.5528 | 1.16E+06 | 152.4599 | 152.1643 | 104.1663 | 1.72474 | 0.70467 | 116.9223 |
| 99 | 131.4656 | 1998.2 | 105.0002 | 1.05E+06 | 145.7051 | 145.7384 | 90.82356 | 1.90452 | 7.85233 | 114.9733 |
| 100 | 93.81805 | 1998.33 | 99.29833 | 641135.7 | 108.7514 | 108.9114 | 109.8757 | 3.11686 | 6.79599 | 76.13452 |
| 101 | 113.3487 | 1998.219 | 103.4742 | 841093.1 | 127.8602 | 127.8322 | 99.96431 | 2.37574 | 9.88491 | 97.72141 |
| 102 | 95.10686 | 1998.191 | 103.0261 | 653539.2 | 109.6317 | 109.6531 | 112.9136 | 3.05749 | 7.6346 | 78.30975 |
| 103 | 98.1211 | 1998.219 | 103.0681 | 682987.5 | 112.7663 | 112.6616 | 110.4975 | 2.9257 | 10.61604 | 82.7734 |
| 104 | 117.0572 | 1998.229 | 100.8559 | 881935.5 | 131.8802 | 131.9167 | 95.15194 | 2.26573 | 7.80422 | 100.1487 |
| 105 | 107.9352 | 1998.321 | 101.1953 | 783109.4 | 122.6034 | 122.7455 | 101.3174 | 2.55178 | 15.22671 | 94.05375 |
| 106 | 120.921 | 1998.265 | 102.8724 | 925453.1 | 135.4516 | 135.4895 | 94.74507 | 2.15923 | 8.59914 | 104.6295 |
| 107 | 128.387 | 1998.151 | 103.0484 | 1.01E+06 | 142.8658 | 142.9298 | 90.74411 | 1.97385 | 3.80274 | 108.5833 |
| 108 | 90.02503 | 1998.267 | 103.4741 | 605287.2 | 104.5584 | 104.5089 | 117.8381 | 3.30135 | 6.42667 | 72.52277 |
| 109 | 138.4501 | 1998.145 | 106.2385 | 1.14E+06 | 152.5984 | 152.5561 | 88.34797 | 1.7603 | 4.1116 | 119.3416 |
| 110 | 94.2906 | 1998.144 | 102.3479 | 645670.5 | 109.033 | 108.9329 | 112.8518 | 3.09468 | 10.4057 | 78.79462 |
| 111 | 102.5567 | 1998.376 | 102.7034 | 727438.3 | 117.0533 | 117.1501 | 106.6895 | 2.74714 | 14.59438 | 88.59277 |
| 112 | 112.3286 | 1998.219 | 100.108 | 830017.9 | 127.217 | 127.2991 | 97.35532 | 2.40744 | 9.6187 | 96.285 |
| 113 | 108.6329 | 1998.291 | 104.4482 | 790472.9 | 122.9386 | 122.9818 | 104.086 | 2.52797 | 5.42906 | 90.50493 |
| 114 | 110.1203 | 1998.099 | 107.9367 | 806279.3 | 124.1481 | 124.0041 | 106.5029 | 2.47817 | 6.83023 | 93.30503 |
| 115 | 98.17177 | 1998.309 | 99.57539 | 683487.8 | 113.1737 | 113.223 | 106.714 | 2.92369 | 8.64966 | 81.59994 |
| 116 | 113.7206 | 1998.295 | 104.2114 | 845147.5 | 128.1207 | 128.1021 | 100.4347 | 2.36443 | 5.80303 | 95.85316 |
| 117 | 106.5373 | 1998.337 | 97.714 | 768453.7 | 121.8932 | 121.8754 | 98.76038 | 2.60047 | 8.04781 | 89.45587 |
| 118 | 112.5271 | 1998.229 | 104.7351 | 832167.6 | 126.8228 | 126.8363 | 101.7236 | 2.40123 | 9.2126 | 96.70049 |

| | | | | | | | | | | |
|---|---|---|---|---|---|---|---|---|---|---|
| 119 | 115.446 | 1998.256 | 102.0846 | 864079.8 | 130.1705 | 130.1269 | 97.30121 | 2.31258 | 7.43125 | 98.44012 |
| 120 | 96.72767 | 1998.246 | 103.13 | 669297.7 | 111.2273 | 111.2597 | 111.689 | 2.98559 | 9.16367 | 80.73822 |
| 121 | 110.4027 | 1998.313 | 98.49471 | 809297.1 | 125.5332 | 125.6191 | 97.00492 | 2.4692 | 10.43668 | 94.58365 |
| 122 | 87.09303 | 1998.087 | 106.8897 | 578248.8 | 101.0768 | 101.1128 | 124.5413 | 3.45541 | 3.27842 | 67.1185 |
| 123 | 95.73785 | 1998.313 | 104.4838 | 659652.9 | 110.0398 | 110.082 | 113.9793 | 3.02934 | 10.39485 | 80.41403 |
| 124 | 100.6632 | 1998.256 | 102.8802 | 708300.8 | 115.1909 | 115.2306 | 108.3073 | 2.8212 | 7.27486 | 83.64116 |
| 125 | 111.3538 | 1998.347 | 103.769 | 819499.1 | 125.7127 | 125.797 | 101.5613 | 2.4385 | 9.7819 | 95.70481 |
| 126 | 120.2284 | 1998.262 | 103.064 | 917579.9 | 134.6657 | 134.7698 | 95.32783 | 2.17775 | 7.34391 | 103.2651 |
| 127 | 116.5785 | 1998.067 | 105.0729 | 876612.2 | 130.8065 | 130.8405 | 99.43098 | 2.27931 | 5.20111 | 98.33485 |
| 128 | 105.9533 | 1998.297 | 102.1306 | 762369.6 | 120.5351 | 120.6278 | 103.6353 | 2.62117 | 10.76776 | 90.58977 |
| 129 | 99.97717 | 1998.269 | 104.5158 | 701426.4 | 114.3216 | 114.3166 | 110.5671 | 2.84887 | 6.50362 | 82.62923 |
| 130 | 90.84846 | 1998.192 | 103.8745 | 612986.4 | 105.2764 | 105.2759 | 117.5488 | 3.25976 | 8.91026 | 74.80238 |
| 131 | 101.5308 | 1998.277 | 104.8636 | 717038.9 | 115.9068 | 115.8228 | 109.7206 | 2.78685 | 5.81251 | 83.73681 |
| 132 | 97.86817 | 1998.185 | 101.0489 | 680492.9 | 112.7187 | 112.699 | 108.5312 | 2.93638 | 5.09703 | 79.10083 |
| 133 | 106.8155 | 1998.264 | 98.70658 | 771360.1 | 121.892 | 121.9988 | 99.57546 | 2.59057 | 8.76833 | 90.2226 |
| 134 | 99.50008 | 1998.285 | 94.84973 | 696664.6 | 115.2487 | 115.301 | 100.6837 | 2.86836 | 10.89853 | 83.54976 |
| 135 | 105.3992 | 1998.29 | 101.9701 | 756618.4 | 120.0306 | 120.0968 | 103.865 | 2.64108 | 8.10742 | 88.76534 |
| 136 | 98.00433 | 1998.338 | 100.144 | 681835.3 | 112.9141 | 112.9703 | 107.4534 | 2.93082 | 11.15211 | 82.63048 |
| 137 | 108.9406 | 1998.199 | 100.7927 | 793731.4 | 123.8075 | 123.8093 | 100.2368 | 2.51748 | 7.46328 | 91.82775 |
| 138 | 109.8519 | 1998.255 | 104.705 | 803416.2 | 124.2082 | 124.1654 | 103.498 | 2.4872 | 4.32299 | 90.7833 |
| 139 | 88.55515 | 1998.296 | 101.2635 | 591658.8 | 103.3507 | 103.3554 | 116.6412 | 3.37745 | 10.46162 | 72.98967 |
| 140 | 99.66752 | 1998.184 | 107.5856 | 698334.1 | 113.4313 | 113.5973 | 114.0663 | 2.86136 | 3.99184 | 80.5839 |
| 141 | 95.00233 | 1998.333 | 101.2625 | 652529 | 109.7682 | 109.803 | 111.0666 | 3.06244 | 7.86468 | 78.16659 |
| 142 | 114.2637 | 1998.213 | 105.3393 | 851085.4 | 128.5239 | 128.4907 | 101.167 | 2.34784 | 6.12885 | 96.7435 |

| | | | | | | | | | | |
|---|---|---|---|---|---|---|---|---|---|---|
| 143 | 94.44689 | 1998.107 | 97.7837 | 647173.6 | 109.641 | 109.7724 | 107.6939 | 3.08744 | 9.76621 | 78.26 |
| 144 | 116.7972 | 1998.278 | 105.7414 | 879042.1 | 130.8964 | 130.9705 | 99.92518 | 2.27325 | 8.68511 | 100.8028 |
| 145 | 104.2824 | 1998.105 | 102.293 | 745089 | 118.9333 | 118.9323 | 104.9969 | 2.6817 | 9.65345 | 88.44977 |
| 146 | 108.6908 | 1998.232 | 107.8073 | 791085.9 | 122.5873 | 122.5922 | 107.3918 | 2.52594 | 5.5903 | 91.02625 |
| 147 | 108.3718 | 1998.356 | 105.3466 | 787713.9 | 122.5492 | 122.5988 | 105.165 | 2.53691 | 4.15694 | 89.20865 |
| 148 | 293.6418 | 1998.214 | 25.72669 | 3.82E+06 | 351.6915 | 351.8949 | 11.66856 | 0.52365 | 14.10877 | 265.5931 |
| 149 | 454.0271 | 1998.24 | 17.53373 | 8.04E+06 | 520.9695 | 539.5012 | 5.47966 | 0.24862 | 2.51E-04 | 224.6998 |
| 150 | 525.9051 | 1998.114 | 19.11335 | 1.04E+07 | 590.673 | 604.3103 | 5.25498 | 0.19241 | 7.32E-04 | 328.6101 |
| 151 | 354.6406 | 1998.114 | 28.19212 | 5.25E+06 | 404.3141 | 407.7968 | 10.89798 | 0.38035 | 0 | 93.82441 |
| 152 | 493.0496 | 1998.231 | 33.01071 | 9.28E+06 | 540.2405 | 538.4492 | 9.60235 | 0.21539 | 0.00131 | 370.2867 |
| 153 | 270.2126 | 1998.34 | 25.42394 | 3.32E+06 | 328.9567 | 329.1632 | 12.36259 | 0.60191 | 13.55791 | 241.4452 |
| 154 | 355.3876 | 1998.338 | 26.35383 | 5.27E+06 | 412.1643 | 412.258 | 10.1691 | 0.37903 | 1.18E+01 | 325.1056 |
| 155 | 343.0894 | 1998.135 | 27.64083 | 4.97E+06 | 397.3726 | 397.3064 | 10.99063 | 0.40244 | 7.67E+00 | 307.8292 |
| 156 | 265.8412 | 1998.215 | 26.06961 | 3.23E+06 | 323.2078 | 323.3281 | 12.85004 | 0.61846 | 8.64E+00 | 231.1092 |
| 157 | 271.0195 | 1998.165 | 30.32273 | 3.34E+06 | 321.1365 | 320.442 | 14.70804 | 0.59887 | 0.00E+00 | 0.00585 |
| 158 | 338.9229 | 1998.137 | 30.96039 | 4.86E+06 | 388.7796 | 387.3268 | 12.43917 | 0.41089 | 0.01762 | 237.4025 |
| 159 | 306.8958 | 1998.066 | 25.72514 | 4.11E+06 | 364.8817 | 365.1481 | 11.24217 | 0.4861 | 1.15E+01 | 275.9106 |
| 160 | 250.3686 | 1998.161 | 25.14705 | 2.92E+06 | 309.7096 | 309.9629 | 13.02815 | 0.68321 | 13.24256 | 221.123 |
| 161 | 529.9387 | 1998.24 | 16.24398 | 1.05E+07 | 601.3103 | 622.1994 | 4.43629 | 0.18986 | 8.60E-05 | 266.8943 |
| 162 | 641.0364 | 1998.215 | 5.1436 | 1.47E+07 | 836.6565 | 932.4009 | 1.18786 | 0.13576 | 0.00184 | 125.6491 |
| 163 | 340.4267 | 1998.231 | 26.04625 | 4.90E+06 | 397.3898 | 397.9657 | 10.42546 | 0.40783 | 14.93338 | 313.3294 |
| 164 | 270.4147 | 1998.404 | 26.16623 | 3.32E+06 | 327.3443 | 327.6947 | 12.71561 | 0.60118 | 10.3277 | 238.2143 |
| 165 | 303.6245 | 1998.282 | 24.79433 | 4.04E+06 | 363.7886 | 364.0702 | 10.93373 | 0.49502 | 1.35E+01 | 274.4242 |
| 166 | 317.6611 | 1998.253 | 25.71278 | 4.36E+06 | 375.8834 | 375.9469 | 10.91427 | 0.45864 | 1.31E+01 | 288.5028 |

| | | | | | | | | | | |
|---|---|---|---|---|---|---|---|---|---|---|
| 167 | 266.6025 | 1998.306 | 26.38688 | 3.25E+06 | 323.487 | 323.4008 | 12.97549 | 0.61555 | 4.04E+00 | 221.7339 |
| 168 | 223.8741 | 1998.421 | 27.09808 | 2.43E+06 | 278.9263 | 279.1849 | 15.393 | 0.82146 | 1.74E-02 | 111.4646 |
| 169 | 245.5451 | 1998.307 | 17.53667 | 2.83E+06 | 317.7087 | 331.0077 | 9.23275 | 0.7056 | 2.17E-03 | 52.94459 |
| 170 | 252.1425 | 1998.317 | 21.30663 | 2.96E+06 | 315.5155 | 322.4839 | 10.97415 | 0.67532 | 0.00765 | 105.1049 |
| 171 | 327.1622 | 1998.285 | 30.86272 | 4.58E+06 | 376.999 | 375.7228 | 12.77736 | 0.43632 | 4.10E-04 | 185.6932 |
| 172 | 195.4505 | 1998.346 | 26.99423 | 1.95E+06 | 251.1863 | 250.972 | 17.12114 | 1.02406 | 6.62E+00 | 157.7543 |
| 173 | 307.926 | 1998.351 | 25.85084 | 4.13E+06 | 365.5733 | 365.9034 | 11.26521 | 0.48343 | 11.52361 | 277.0744 |
| 174 | 505.896 | 1998.414 | 21.91288 | 9.70E+06 | 562.969 | 574.2946 | 6.23261 | 0.20595 | 0.00E+00 | 118.304 |
| 175 | 396.8124 | 1998.252 | 26.82445 | 6.37E+06 | 452.2276 | 452.6826 | 9.4178 | 0.31378 | 10.27795 | 364.9423 |
| 176 | 278.197 | 1998.232 | 26.30461 | 3.49E+06 | 335.2805 | 335.1708 | 12.4835 | 0.5733 | 5.39754 | 237.1822 |
| 177 | 289.4185 | 1998.243 | 29.87733 | 3.72E+06 | 341.1027 | 339.5797 | 13.71698 | 0.53655 | 3.58E-02 | 192.9974 |
| 178 | 260.3736 | 1998.104 | 29.74022 | 3.12E+06 | 311.6872 | 310.7625 | 14.91499 | 0.64019 | 1.43E-04 | 103.5279 |
| 179 | 228.3023 | 1998.275 | 29.66284 | 2.51E+06 | 280.0636 | 278.827 | 16.5817 | 0.79546 | 6.51E-04 | 87.33653 |
| 180 | 231.0082 | 1998.194 | 28.81269 | 2.56E+06 | 283.2711 | 283.0216 | 15.95148 | 0.78019 | 6.00E-06 | 35.97189 |
| 181 | 229.2132 | 1998.171 | 28.3118 | 2.53E+06 | 282.0698 | 282.1462 | 15.77484 | 0.79024 | 3.00E-06 | 23.99028 |
| 182 | 228.1312 | 1998.345 | 30.88808 | 2.51E+06 | 278.3046 | 276.6534 | 17.27723 | 0.79647 | 0 | 0.00575 |
| 183 | 238.2233 | 1998.34 | 28.94298 | 2.69E+06 | 290.6521 | 290.0064 | 15.62337 | 0.74176 | 2.00E-05 | 56.95354 |
| 184 | 236.8498 | 1998.1 | 26.05859 | 2.67E+06 | 293.0879 | 294.3577 | 14.13353 | 0.74877 | 0.03888 | 131.1203 |
| 185 | 208.4951 | 1998.332 | 26.55458 | 2.17E+06 | 265.1006 | 264.9354 | 15.9851 | 0.92246 | 6.71946 | 170.6639 |
| 186 | 395.8912 | 1998.341 | 32.53234 | 6.34E+06 | 443.9891 | 441.9609 | 11.44464 | 0.31505 | 1.05E-03 | 269.5031 |
| 187 | 248.7393 | 1998.385 | 28.05925 | 2.89E+06 | 302.837 | 302.1544 | 14.61567 | 0.69071 | 1.56E+00 | 193.4381 |
| 188 | 228.6509 | 1998.39 | 27.60505 | 2.52E+06 | 283.8934 | 282.945 | 15.41208 | 0.79352 | 0.37239 | 154.836 |
| 189 | 238.4924 | 1998.251 | 27.84175 | 2.70E+06 | 290.9106 | 292.3212 | 15.01496 | 0.74035 | 1.00E-06 | 14.21226 |
| 190 | 236.6835 | 1998.26 | 29.73272 | 2.67E+06 | 287.9986 | 287.089 | 16.13561 | 0.74969 | 7.50E-05 | 73.02815 |

| | | | | | | | | | | |
|---|---|---|---|---|---|---|---|---|---|---|
| 191 | 227.8948 | 1998.167 | 28.83825 | 2.50E+06 | 280.0151 | 279.8614 | 16.14438 | 0.79775 | 4.00E-06 | 29.85024 |
| 192 | 237.1817 | 1998.125 | 25.53071 | 2.67E+06 | 293.4 | 295.8793 | 13.83126 | 0.74705 | 1.16E-04 | 57.63698 |
| 193 | 224.9768 | 1998.209 | 29.24485 | 2.45E+06 | 277.4724 | 276.2219 | 16.54579 | 0.81479 | 1.56E-04 | 66.9608 |
| 194 | 213.5034 | 1998.354 | 28.16571 | 2.25E+06 | 267.1311 | 266.7159 | 16.63096 | 0.88756 | 0.00454 | 88.77591 |
| 195 | 224.5691 | 1998.167 | 28.99247 | 2.45E+06 | 277.1465 | 276.2592 | 16.42738 | 0.8172 | 4.70E-05 | 52.41545 |
| 196 | 232.6713 | 1998.266 | 29.47169 | 2.59E+06 | 284.3536 | 283.5235 | 16.22045 | 0.77108 | 1.01E-04 | 70.96527 |
| 197 | 229.8881 | 1998.126 | 29.69733 | 2.54E+06 | 281.6372 | 280.3504 | 16.50697 | 0.78642 | 1.80E-03 | 100.0341 |
| 198 | 250.5131 | 1998.114 | 28.41723 | 2.93E+06 | 302.8275 | 303.2482 | 14.71533 | 0.68254 | 4.00E-06 | 49.71614 |
| 199 | 227.2089 | 1998.387 | 29.99032 | 2.49E+06 | 278.6891 | 277.1847 | 16.83087 | 0.80179 | 1.41E-03 | 95.72482 |
| 200 | 217.218 | 1998.376 | 29.10821 | 2.32E+06 | 270.3463 | 268.708 | 16.94762 | 0.86297 | 0.03846 | 119.7298 |
| 201 | 241.5593 | 1998.129 | 28.75295 | 2.76E+06 | 294.0084 | 293.6791 | 15.34387 | 0.72487 | 1.70E-05 | 57.30756 |
| 202 | 216.3448 | 1998.326 | 28.45513 | 2.30E+06 | 269.528 | 269.0153 | 16.62188 | 0.86863 | 0.0038 | 90.58897 |
| 203 | 222.3824 | 1998.307 | 29.36117 | 2.41E+06 | 274.6066 | 273.4271 | 16.77 | 0.83045 | 3.97E-04 | 74.92526 |
| 204 | 187.3446 | 1998.344 | 27.61479 | 1.82E+06 | 242.7571 | 241.6184 | 18.12078 | 1.09615 | 0.24767 | 108.5236 |
| 205 | 196.7105 | 1998.139 | 28.40419 | 1.97E+06 | 251.0274 | 249.4704 | 17.9224 | 1.01341 | 0.07738 | 105.6381 |
| 206 | 207.4774 | 1998.32 | 29.34491 | 2.15E+06 | 259.9695 | 258.5506 | 17.73508 | 0.92981 | 0.00198 | 77.46952 |
| 207 | 232.4994 | 1998.309 | 28.52389 | 2.59E+06 | 284.9191 | 285.0425 | 15.70834 | 0.77203 | 3.00E-06 | 26.97923 |
| 208 | 211.8681 | 1998.385 | 29.14196 | 2.22E+06 | 264.6787 | 263.2988 | 17.31538 | 0.89874 | 0.00105 | 74.13711 |
| 209 | 212.4216 | 1998.208 | 28.03662 | 2.23E+06 | 266.2552 | 265.8751 | 16.6233 | 0.89486 | 5.89E-03 | 90.26245 |
| 210 | 228.0124 | 1998.121 | 29.37767 | 2.51E+06 | 279.6252 | 279.0236 | 16.43941 | 0.79706 | 4.00E-05 | 56.1514 |
| 211 | 212.5674 | 1998.293 | 29.46299 | 2.24E+06 | 263.9755 | 263.4353 | 17.45927 | 0.8939 | 2.10E-05 | 34.11232 |
| 212 | 205.9717 | 1998.323 | 29.95065 | 2.12E+06 | 257.5656 | 256.0121 | 18.20839 | 0.94086 | 0.00149 | 74.91327 |
| 213 | 215.2021 | 1998.275 | 29.26517 | 2.28E+06 | 267.2335 | 266.4133 | 17.169 | 0.87614 | 7.10E-05 | 48.87595 |
| 214 | 209.6592 | 1998.39 | 29.86793 | 2.19E+06 | 261.1346 | 259.8399 | 17.89854 | 0.91419 | 2.68E-04 | 59.98104 |

| | | | | | | | | | | |
|---|---|---|---|---|---|---|---|---|---|---|
| 215 | 204.9087 | 1998.276 | 29.22701 | 2.11E+06 | 257.7238 | 256.1868 | 17.8431 | 0.94876 | 0.03113 | 105.3158 |
| 216 | 205.6897 | 1998.32 | 29.11981 | 2.12E+06 | 258.0557 | 257.1578 | 17.72295 | 0.94295 | 3.90E-04 | 57.08301 |
| 217 | 207.7327 | 1998.345 | 28.98971 | 2.15E+06 | 260.9964 | 259.4324 | 17.50294 | 0.92797 | 0.02589 | 105.4178 |
| 218 | 198.6491 | 1998.148 | 29.39355 | 2.00E+06 | 251.1886 | 249.6335 | 18.40056 | 0.99751 | 0.00926 | 85.87056 |
| 219 | 220.7561 | 1998.151 | 28.68292 | 2.38E+06 | 273.4589 | 273.0037 | 16.48119 | 0.84041 | 2.53E-04 | 65.59942 |
| 220 | 205.4716 | 1998.119 | 29.47214 | 2.12E+06 | 257.9765 | 256.3192 | 17.95281 | 0.94448 | 0.00234 | 77.73159 |
| 221 | 218.5553 | 1998.14 | 28.45452 | 2.34E+06 | 271.6959 | 271.222 | 16.48427 | 0.85427 | 2.85E-04 | 63.72338 |
| 222 | 211.6052 | 1998.21 | 28.93193 | 2.22E+06 | 263.5967 | 263.4047 | 17.20795 | 0.90048 | 4.00E-06 | 12.2722 |
| 223 | 217.6835 | 1998.215 | 29.12523 | 2.32E+06 | 270.0094 | 269.1393 | 16.92795 | 0.85989 | 8.10E-05 | 52.0365 |
| 224 | 216.9577 | 1998.275 | 29.38394 | 2.31E+06 | 269.5953 | 267.962 | 17.1249 | 0.86461 | 0.00833 | 102.9678 |
| 225 | 209.3274 | 1998.335 | 29.66092 | 2.18E+06 | 261.6002 | 259.8569 | 17.79737 | 0.91652 | 0.0034 | 86.30506 |
| 226 | 213.5394 | 1998.283 | 28.86753 | 2.25E+06 | 266.6303 | 265.4563 | 17.04302 | 0.88728 | 0.00234 | 83.73524 |
| 227 | 212.4511 | 1998.28 | 28.48089 | 2.23E+06 | 265.6708 | 265.0727 | 16.88481 | 0.89469 | 0.00465 | 89.064 |
| 228 | 202.6187 | 1998.397 | 27.54918 | 2.07E+06 | 258.2065 | 257.0231 | 16.97249 | 0.96624 | 0.28594 | 125.4324 |
| 229 | 181.7557 | 1998.154 | 28.41997 | 1.74E+06 | 235.9955 | 234.4868 | 19.10607 | 1.15042 | 0.06636 | 88.91187 |
| 230 | 172.2031 | 1998.102 | 26.95337 | 1.59E+06 | 228.707 | 227.8019 | 18.91439 | 1.25345 | 0.69505 | 105.0658 |
| 231 | 205.6106 | 1998.385 | 29.77548 | 2.12E+06 | 257.0086 | 255.9469 | 18.12765 | 0.94357 | 4.17E-04 | 60.27947 |
| 232 | 204.7054 | 1998.104 | 28.96345 | 2.10E+06 | 257.9963 | 256.4457 | 17.69642 | 0.95021 | 0.00802 | 89.03899 |
| 233 | 216.3965 | 1998.346 | 29.34951 | 2.30E+06 | 268.0572 | 267.4624 | 17.14099 | 0.8683 | 1.30E-05 | 32.52473 |
| 234 | 215.9291 | 1998.124 | 27.29113 | 2.29E+06 | 269.8613 | 270.8405 | 15.96695 | 0.87127 | 6.50E-05 | 39.07075 |

Table S2 The original nanoindentation data for line scanning.

| Point | hc(nm) | Pmax(μ N) | S(μ N/nm) | A(nm^2) | hmax(nm) | heff(nm) | Er(GPa) | H(GPa) | A | hf(nm) |
|---|---|---|---|---|---|---|---|---|---|---|
| 1 | 148.1146 | 2998.392 | 133.6587 | 1.18E+06 | 165.1429 | 164.9395 | 109.026 | 2.54147 | 10.87171 | 129.5821 |
| 2 | 140.7704 | 2998.333 | 133.7154 | 1.09E+06 | 157.6671 | 157.5878 | 113.3271 | 2.74356 | 10.88232 | 122.2476 |
| 3 | 157.5038 | 2998.593 | 132.1293 | 1.30E+06 | 174.5886 | 174.5246 | 102.8616 | 2.31503 | 14.23265 | 140.1876 |
| 4 | 130.5793 | 2998.484 | 142.3889 | 977221 | 146.4252 | 146.3731 | 127.6188 | 3.06838 | 8.31648 | 111.4726 |
| 5 | 144.7465 | 2998.692 | 148.9575 | 1.14E+06 | 159.9113 | 159.8449 | 123.6319 | 2.63147 | 11.27368 | 127.5098 |
| 6 | 156.6927 | 2998.37 | 147.9602 | 1.29E+06 | 171.9858 | 171.8912 | 115.6406 | 2.33317 | 14.27716 | 140.4623 |
| 7 | 156.7575 | 2998.433 | 144.1926 | 1.29E+06 | 172.3822 | 172.3535 | 112.6604 | 2.33175 | 13.47496 | 140.0199 |
| 8 | 116.8864 | 2998.582 | 136.9925 | 831035.5 | 133.3243 | 133.3029 | 133.144 | 3.60825 | 16.60969 | 100.671 |
| 9 | 115.426 | 2998.612 | 128.2162 | 816069.8 | 133.0577 | 132.9663 | 125.7518 | 3.67446 | 18.14797 | 99.06577 |
| 10 | 94.95663 | 2998.406 | 122.5191 | 619138.7 | 113.2964 | 113.3114 | 137.9573 | 4.84287 | 4.78453 | 71.17742 |
| 11 | 116.8604 | 2998.573 | 219.1652 | 830767.8 | 126.5553 | 127.1217 | 213.0426 | 3.6094 | 2.00E-06 | 58.7664 |
| 12 | 222.7723 | 2998.484 | 254.0171 | 2.23E+06 | 231.4918 | 231.6255 | 150.675 | 1.34397 | 5.04648 | 207.8358 |
| 13 | 321.595 | 2998.51 | 224.8035 | 4.08E+06 | 331.5557 | 331.5988 | 98.60339 | 0.73487 | 13.52858 | 308.6169 |
| 14 | 343.4169 | 2998.516 | 213.004 | 4.56E+06 | 353.972 | 353.9749 | 88.4006 | 0.65792 | 15.34034 | 330.4572 |
| 15 | 336.0757 | 2998.296 | 206.6359 | 4.39E+06 | 346.8084 | 346.9583 | 87.33693 | 0.68232 | 13.84477 | 322.5379 |
| 16 | 356.0238 | 2998.652 | 190.1024 | 4.84E+06 | 367.7345 | 367.8542 | 76.52346 | 0.61897 | 14.82438 | 342.0791 |
| 17 | 314.0164 | 2998.27 | 162.7459 | 3.92E+06 | 327.9621 | 327.8336 | 72.82547 | 0.7648 | 4.55239 | 293.9062 |
| 18 | 210.9629 | 2998.338 | 93.30538 | 2.04E+06 | 235.1112 | 235.0639 | 57.81321 | 1.4664 | 1.85502 | 176.6882 |
| 19 | 271.5921 | 2998.339 | 102.6535 | 3.08E+06 | 293.4425 | 293.4984 | 51.8215 | 0.97338 | 2.98911 | 242.2287 |

| | | | | | | | | | | |
|---|---|---|---|---|---|---|---|---|---|---|
| 20 | 290.0705 | 2998.584 | 104.9518 | 3.43E+06 | 311.571 | 311.4988 | 50.17483 | 0.87305 | 0.40497 | 249.756 |
| 21 | 206.8762 | 2998.54 | 108.6651 | 1.98E+06 | 227.6492 | 227.5719 | 68.38792 | 1.51293 | 0.89364 | 171.8506 |
| 22 | 274.6453 | 2998.511 | 100.2116 | 3.14E+06 | 297.1391 | 297.0866 | 50.12469 | 0.95566 | 1.0804 | 238.7449 |
| 23 | 252.6377 | 2998.389 | 101.7215 | 2.74E+06 | 274.6798 | 274.745 | 54.49051 | 1.09606 | 0.77174 | 215.1457 |
| 24 | 259.9581 | 2998.523 | 97.50361 | 2.87E+06 | 283.1137 | 283.0228 | 51.02467 | 1.04606 | 2.3262 | 228.0552 |
| 25 | 258.9459 | 2998.474 | 97.02531 | 2.85E+06 | 282.1815 | 282.124 | 50.93693 | 1.05275 | 1.32698 | 223.4992 |
| 26 | 226.4251 | 2998.644 | 105.5923 | 2.29E+06 | 247.8099 | 247.7239 | 61.81978 | 1.30932 | 0.97883 | 191.2184 |
| 27 | 286.5205 | 2998.597 | 105.158 | 3.37E+06 | 308.0743 | 307.9069 | 50.78953 | 0.89107 | 0.10842 | 239.0043 |
| 28 | 244.9234 | 2998.665 | 109.7519 | 2.60E+06 | 265.629 | 265.4151 | 60.29736 | 1.15301 | 0.19248 | 201.8822 |
| 29 | 285.6231 | 2998.519 | 102.9316 | 3.35E+06 | 307.4898 | 307.4714 | 49.84367 | 0.89569 | 2.26397 | 254.6744 |
| 30 | 277.5923 | 2998.507 | 95.1772 | 3.19E+06 | 301.1358 | 301.2206 | 47.18901 | 0.93897 | 2.54997 | 245.7564 |
| 31 | 245.3367 | 2998.431 | 102.8165 | 2.61E+06 | 267.5105 | 267.2089 | 56.40967 | 1.14976 | 3.0012 | 216.0264 |
| 32 | 284.5602 | 2998.533 | 102.0489 | 3.33E+06 | 306.6913 | 306.5977 | 49.56909 | 0.90125 | 2.18645 | 253.2302 |
| 33 | 273.3673 | 2998.518 | 99.9849 | 3.11E+06 | 295.9919 | 295.8596 | 50.20406 | 0.96304 | 1.71561 | 240.1609 |
| 34 | 252.5571 | 2998.69 | 97.07552 | 2.73E+06 | 275.6406 | 275.7248 | 52.01531 | 1.09674 | 0.76895 | 213.8147 |
| 35 | 258.3629 | 2998.351 | 99.48759 | 2.84E+06 | 280.9155 | 280.9663 | 52.3261 | 1.0566 | 1.40711 | 223.8609 |
| 36 | 258.9191 | 2998.731 | 98.3693 | 2.85E+06 | 281.814 | 281.7824 | 51.64688 | 1.05302 | 0.80584 | 220.8071 |
| 37 | 237.0475 | 2998.58 | 101.1666 | 2.47E+06 | 259.3049 | 259.2775 | 57.07527 | 1.21582 | 1.44204 | 203.0735 |
| 38 | 204.8151 | 2998.389 | 107.7375 | 1950634 | 225.8488 | 225.6879 | 68.3461 | 1.537136 | 0.415937 | 165.382 |
| 39 | 271.5921 | 2998.339 | 102.6535 | 3080331 | 293.4425 | 293.4984 | 51.8215 | 0.973382 | 2.989111 | 242.2287 |
| 40 | 290.0705 | 2998.584 | 104.9518 | 3434612 | 311.571 | 311.4988 | 50.17483 | 0.873049 | 0.404965 | 249.756 |
| 41 | 206.8762 | 2998.54 | 108.6651 | 1981943 | 227.6492 | 227.5719 | 68.38793 | 1.512929 | 0.893644 | 171.8506 |
| 42 | 274.6453 | 2998.511 | 100.2115 | 3137630 | 297.139 | 297.0866 | 50.12469 | 0.955661 | 1.080404 | 238.7449 |
| 43 | 252.6377 | 2998.389 | 101.7214 | 2735602 | 274.6798 | 274.745 | 54.49051 | 1.096062 | 0.771741 | 215.1457 |

| | | | | | | | | | | |
|---|---|---|---|---|---|---|---|---|---|---|
| 44 | 259.9581 | 2998.523 | 97.50361 | 2866492 | 283.1137 | 283.0228 | 51.02467 | 1.04606 | 2.326203 | 228.0552 |
| 45 | 258.9459 | 2998.474 | 97.02531 | 2848225 | 282.1815 | 282.1239 | 50.93693 | 1.052752 | 1.326982 | 223.4992 |
| 46 | 226.4251 | 2998.644 | 105.5923 | 2290231 | 247.8099 | 247.7239 | 61.81978 | 1.309319 | 0.978828 | 191.2184 |
| 47 | 286.5205 | 2998.597 | 105.158 | 3365158 | 308.0743 | 307.9069 | 50.78953 | 0.891072 | 0.108416 | 239.0042 |
| 48 | 244.9234 | 2998.665 | 109.7519 | 2600740 | 265.629 | 265.415 | 60.29736 | 1.153005 | 0.192478 | 201.8822 |
| 49 | 285.623 | 2998.519 | 102.9316 | 3347704 | 307.4898 | 307.4714 | 49.84367 | 0.895694 | 2.263972 | 254.6744 |
| 50 | 277.5922 | 2998.507 | 95.1772 | 3193402 | 301.1358 | 301.2206 | 47.18902 | 0.938969 | 2.549975 | 245.7564 |
| 51 | 245.3367 | 2998.431 | 102.8165 | 2607886 | 267.5105 | 267.2089 | 56.40967 | 1.149756 | 3.001197 | 216.0264 |
| 52 | 284.5602 | 2998.533 | 102.0489 | 3327088 | 306.6913 | 306.5977 | 49.56909 | 0.901248 | 2.18645 | 253.2302 |
| 53 | 273.3673 | 2998.518 | 99.9849 | 3113586 | 295.9919 | 295.8596 | 50.20406 | 0.963043 | 1.715605 | 240.1609 |
| 54 | 252.5571 | 2998.69 | 97.07552 | 2734177 | 275.6406 | 275.7248 | 52.01531 | 1.096743 | 0.768948 | 213.8147 |
| 55 | 258.3629 | 2998.351 | 99.48759 | 2837727 | 280.9155 | 280.9663 | 52.3261 | 1.056603 | 1.40711 | 223.8609 |
| 56 | 258.9191 | 2998.731 | 98.3693 | 2847741 | 281.814 | 281.7824 | 51.64688 | 1.053021 | 0.805839 | 220.807 |
| 57 | 237.0475 | 2998.58 | 101.1666 | 2466310 | 259.3049 | 259.2775 | 57.07527 | 1.215816 | 1.442035 | 203.0735 |
